# Emergence of Pituitary Adenoma in a Child during Surveillance: Clinical Challenges and the Family Members' View in an *AIP* Mutation-Positive Family

**DOI:** 10.1155/2018/8581626

**Published:** 2018-04-04

**Authors:** Pedro Marques, Sayka Barry, Amy Ronaldson, Arla Ogilvie, Helen L. Storr, Peter J. Goadsby, Michael Powell, Mary N. Dang, Harvinder S. Chahal, Jane Evanson, Ajith V. Kumar, Joan Grieve, Márta Korbonits

**Affiliations:** ^1^Centre for Endocrinology, William Harvey Research Institute, Barts and the London School of Medicine and Dentistry, Queen Mary University of London, London, UK; ^2^West Hertfordshire Hospitals NHS Trust, Watford, UK; ^3^Basic & Clinical Neuroscience and NIHR-Wellcome Trust King's Clinical Research Facility, King's College London, London, UK; ^4^The National Hospital for Neurology and Neurosurgery, UCLH, NHS Trust, London, UK; ^5^Department of Endocrinology, Imperial College Healthcare NHS Trust, London, UK; ^6^Department of Radiology, Barts and the London School of Medicine, Queen Mary University of London, London EC1M 6BQ, UK; ^7^North East Thames Regional Genetics Service, Great Ormond Street Hospital, London, UK

## Abstract

**Introduction:**

Germline aryl hydrocarbon receptor-interacting protein (*AIP*) mutations are responsible for 15–30% of familial isolated pituitary adenomas (FIPAs). We report a FIPA kindred with a heterozygous deletion in *AIP*, aiming to highlight the indications and benefits of genetic screening, variability in clinical presentations, and management challenges in this setting.

**Patients:**

An 18-year-old male was diagnosed with a clinically nonfunctioning pituitary adenoma (NFPA). Two years later, his brother was diagnosed with a somatolactotrophinoma, and a small Rathke's cleft cyst and a microadenoma were detected on screening in their 17-year-old sister. Following amenorrhoea, their maternal cousin was diagnosed with hyperprolactinaemia and two distinct pituitary microadenomas. A 12-year-old niece developed headache and her MRI showed a microadenoma, not seen on a pituitary MRI scan 3 years earlier.

**Discussion:**

Out of the 14 members harbouring germline *AIP* mutations in this kindred, 5 have pituitary adenoma. Affected members had different features and courses of disease. Bulky pituitary and not fully suppressed GH on OGTT can be challenging in the evaluation of females in teenage years. Multiple pituitary adenomas with different secretory profiles may arise in the pituitary of these patients. Small, stable NFPAs can be present in mutation carriers, similar to incidentalomas in the general population. Genetic screening and baseline review, with follow-up of younger subjects, are recommended in *AIP* mutation-positive families.

## 1. Introduction

Pituitary adenomas (PAs) are benign tumours with a high prevalence in autopsy and radiological studies, ranging from 14.4 up to 22.5% [1]. However, clinically relevant PAs are significantly less common, affecting 1 : 1064 up to 1 : 1470 of the general population [2–6]. The great majority of PAs occur sporadically, but around 5% are familial in origin, which either occur as part of a syndrome, such as in multiple endocrine neoplasia type 1 (MEN1) and MEN4 or in Carney complex, or can be isolated to the pituitary as in familial isolated pituitary adenomas (FIPAs). FIPA kindreds are recognised when 2 or more family members display PAs in the absence of other syndromic features.

In 2006, loss-of-function mutations in the aryl hydrocarbon receptor-interacting protein (*AIP*) gene were recognised as predisposing for PAs [7]. The gene encoding the AIP protein is located on chromosome 11q13.3, and to date, over 70 *AIP* mutations have been described. Truncating mutations account for the majority of *AIP* mutations [8–10]. Germline heterozygous *AIP* mutations are responsible for 20% of FIPA [10] and can be identified in 3-4% of unselected sporadic PAs [11, 12], 20.5% of childhood-onset PAs, 11.7% of pituitary macroadenomas in young patients (<30 years) [13], and around 7% of PAs diagnosed in patients under the age of 40 with no known family history of pituitary disease [11]. Growth hormone- (GH-) secreting and GH- and prolactin-cosecreting PAs dominate, but prolactinomas, nonfunctioning PA (NFPA), and few ACTH- and TSH-secreting PAs were also described. PAs in patients with *AIP* mutations present earlier in life and are larger, more invasive, and less responsive to treatment [8, 10].

In this paper, we describe in detail a large four-generation FIPA kindred with a heterozygous *AIP* gene deletion, discussing a variety of clinical scenarios and common management challenges and highlighting the genetic screening benefits in this setting. Taking together our clinical experience and published data on this condition, we aim to answer relevant questions in order to aid clinicians in the recognition, diagnosis, and management of this condition. Moreover, we give voice to patients and carriers by including a section with their own comments regarding their condition; in our opinion, this is helpful for understanding the perspectives and needs in this setting of patients.

## 2. Case 1 (Proband)

**Figure 1:**
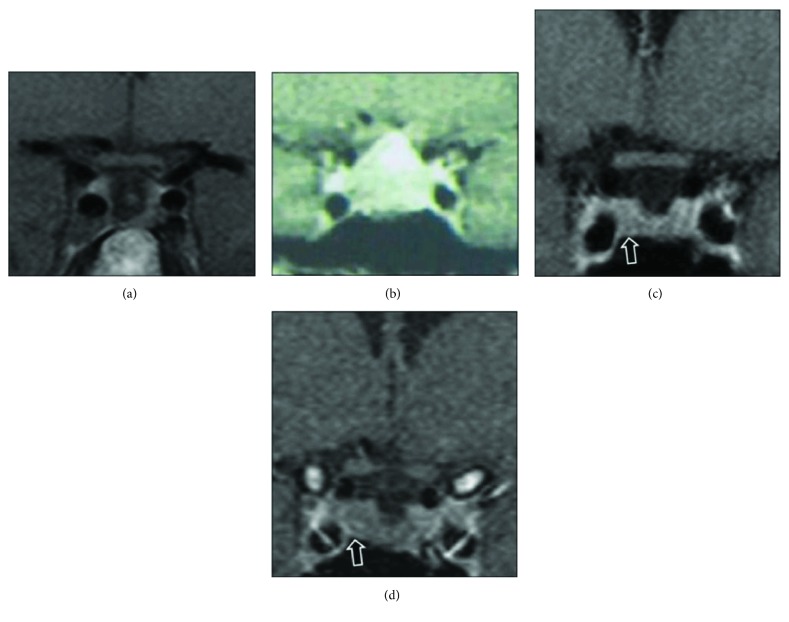
(a) The proband's (case 1) last MRI scan showing a visible thin rim of tissue around the pituitary fossa walls but no recurrent tumour. (b) A pituitary MRI scan of the proband's brother (case 2) at the time of diagnosis, showing a pituitary macroadenoma impinging the optic chiasma. (c) Case 2's follow-up MRI scan 7 years after the original surgery shows a small 5 mm region with reduced enhancement in the right side of the pituitary representing a remnant of the pituitary adenoma, which has recently grown slightly (d) and was operated.

The proband, an 18-year-old male, presented in 2000 with a 3-month history of lethargy, daytime sleepiness, exhaustion, loss of appetite, and headaches, few months after his mother's death due to a spinal ependymoma. These complaints were initially attributed to grief, but their worsening lead to an endocrine investigation, which revealed secondary adrenal failure (9 am serum cortisol: 68 nmol/L, undetectable ACTH), secondary hypothyroidism (FT4 < 5.1 pmol/L [9.4–24.0], TSH: 1.56 mU/L), hyperprolactinaemia (prolactin: 1466 mU/L [45–375]), “borderline” secondary hypogonadism (LH: 1.3 mU/L [0.8–6.1], FSH: 2.3 mU/L [1.6–11.0], and morning total testosterone: 13.0 nmol/L [13–40]), and normal IGF-1 close to the upper limit of the reference range (61.9 nmol/L [29–64]). Basal GH or oral glucose tolerance test (OGTT) were not performed. The remaining blood tests, including serum calcium, were normal. His pituitary MRI demonstrated a pituitary macroadenoma with cavernous sinus invasion and suprasellar extension, impinging and displacing the optic chiasma. He had normal visual fields, normal height (178.2 cm, midparental height: 174.5 cm), and no symptoms of acromegaly. He was diagnosed with a clinically nonfunctioning pituitary adenoma, although an IGF-1 at the upper limit of the reference range, in the presence of a macroadenoma with partial hypopituitarism, raises the possibility of a poorly secreting somatotropinoma. He underwent transsphenoidal surgery (TSS), and the histology showed a chromophobe adenoma with scattered single GH-positive cells and a few clusters of prolactin-positive cells, with the Ki67 index estimated at 5–10%. Seventeen years after the diagnosis, he is well on thyroxine replacement, displaying normal IGF-1 and prolactin, and his recent MRI scan showed no recurrent tumour growth, with a visible thin rim of tissue around the walls of his large pituitary fossa that is stable since his first postoperative scan (Figure 1(a)).

## 3. Case 2

The proband's brother presented in 2002 at the age of 18 years with a 2-month history of frontal headaches and lethargy. Clinical examination revealed tall stature (184.5 cm, midparental height: 174.5 cm). GH excess was documented by an elevated IGF-1 (75.3 nmol/L [29–64]) and a random GH (54 *μ*g/L); OGTT GH nadir was 0.4 *μ*g/L (Table 1). Hyperprolactinaemia (927 mU/L [45–375]) and secondary hypothyroidism (FT4: 7.3 pmol/L [9.4–24.0], TSH: 0.7 mU/L) were also noted, and secondary adrenal failure was excluded by a short Synacthen test (baseline cortisol: 223 nmol/L, peak: 467 nmol/L). Pituitary MRI showed a macroadenoma with suprasellar extension and impingement on the optic chiasm, but no cavernous sinus invasion (Figure 1(b)). A small left upper temporal field deficiency was detected. He underwent TSS, and the histology showed a sparsely granulated somatotroph adenoma with strong expression of GH and prolactin and 10–20% LH- and FSH-positive cells. For the next 13 years after TSS, he had ongoing headaches, but no acromegaly-related complaints. A small 5 mm region with reduced enhancement was visible in the pituitary fossa, possibly representing adenoma remnant (Figure 1(c)), unchanged over those years. He was eupituitary, and during follow-up, his somatotrope axis evaluation was consistent with mild and intermittent biochemical evidence of GH excess (IGF-1 ranging between 168 and 398 *μ*g/L and incomplete GH suppression on OGTT (Table 1)). Considering that he was asymptomatic and reluctant to have medical therapy, a watchful waiting approach was taken. However, since 2015, he became sweaty with occasional joint pain, and his IGF-1 raised at 1.4 × ULN, with an OGTT GH nadir of 2.53 *μ*g/L and a mean GH of 2.59 *μ*g/L on a day curve (Table 1). Moreover, a minimal increase in the remnant was reported on his last MRI (Figure 1(d)). He was commenced on lanreotide (120 mg/every 4 weeks) but developed significant gastrointestinal side effects, particularly nausea and diarrhoea. As biochemical response was unsatisfactory, medication was stopped and TSS was planned. Postoperative MRI showed no residual tumour. His headache significantly improved. He is now on hydrocortisone, thyroxine, and testosterone replacement. The histology of the excised remnant tumoural lesion was similar to that of the first operation, with Ki67 less than 3%.

Headaches were always his main complaint and affected considerably his quality of life. He was reviewed by a headache specialist in a clinic and also by an ear, nose, and throat specialist who excluded sinusitis as a cause of his headaches. The headaches occurred daily and were not associated with migraine-like symptoms. They were triggered by exercise or submersion, leading him to quit diving in summer. He reported two different types of headaches: dull daily headache and the other a sharp and short-lasting (20 seconds to 1 minute) pain in the right side of his head, associated with dizziness, but no nausea, vomiting, or photophobia. His sharp right-sided headaches were diagnosed as paroxysmal hemicrania, a form of trigeminal autonomic cephalalgia [14], ipsilateral to the remnant, despite the absence of cavernous sinus invasion. Short-acting octreotide (100 *μ*g three times a day for a six weeks) and later cabergoline (250 *μ*g three times a week) were tried in 2009 but have not changed his headache considerably, based on his headache diary. On short-acting octreotide, none of the GH measurements from a GH day curve were undetectable, and his IGF-1 was 266 ng/mL (Table 1). Headaches remained significantly improved after the second operation.

## 4. Case 3

**Figure 2:**
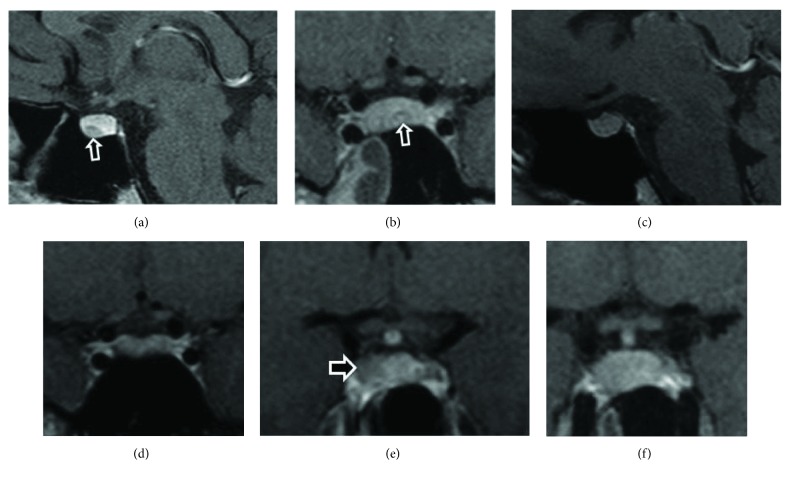
Pituitary MRI scans of the proband's sister (case 3), showing a Rathke's cleft cyst when first screened at the age of 17 (a, b), which completely disappeared in the following 5 years (c, d). A 4 mm microadenoma became visible in the right side (e) of her bulky pituitary (f).

Following the genetic diagnosis, the proband's 17-year-old carrier sister reported occasional headache but no symptoms of GH excess. She displayed normal IGF-1 and OGTT GH nadir consistently lower than 0.4 *μ*g/L (Table 2), although not undetectable. She had no hyperprolactinaemic symptoms, and her prolactin levels have been normal, apart from the last evaluation that is slightly elevated (601 mU/L, normal < 495). Her pituitary MRI when she was first screened showed a large bulky pituitary with a height of 9.2 mm and an area of 4.4 mm of reduced enhancement in the midline, faintly hyperintense before the contrast, suggestive of a Rathke's cleft cyst, which regressed completely over the period of 5 years. In addition, a 4 mm adenoma was visible in the dorsal aspect of the right side of her pituitary, which has been stable over the years (Figure 2).

## 5. Case 4

**Figure 3:**
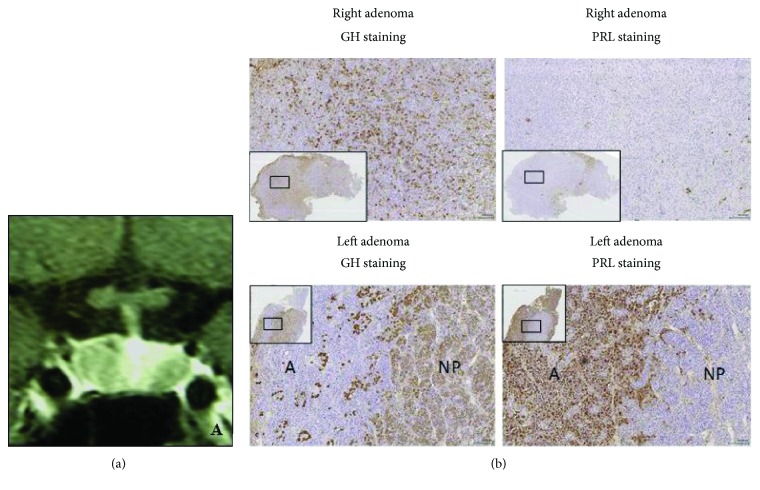
(a) Pituitary MRI of the proband's second cousin (case 4) showing right 6 mm and left 4 mm PAs. (b) Immunostaining of the right (upper 2 panels) and left (lower 2 panels) PAs. The right adenoma shows strong GH and scattered prolactin staining. The lower panels show the left adenoma containing adenoma tissue (A), which stains strongly for prolactin and shows only scattered GH staining, and normal pituitary tissue (NP) which is visible in many GH-stained cells.

**Figure 4:**
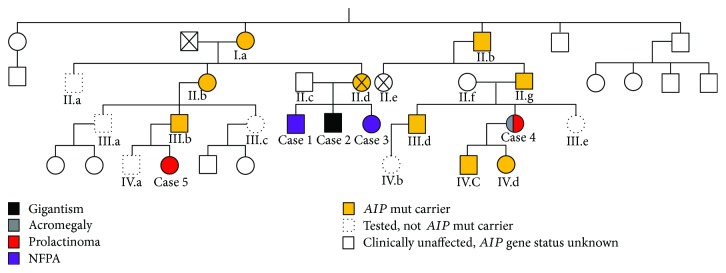
Pedigree tree.

The proband's maternal second cousin presented in 2003, at an age of 27 years, with secondary amenorrhoea after cessation of oral contraceptives. Hyperprolactinaemia was found (1232 mU/L), and bromocriptine was started. On bromocriptine, her menses became regular and her prolactin normalised (65 mU/L), but her IGF-1 was raised at 97 nmol/L [23.0–49.6], and her GH was not suppressed on OGTT (GH nadir 6.5 *μ*g/L) with little clinical features. Her MRI identified two pituitary microadenomas, a 6 mm adenoma on the right and a 4 mm adenoma on the left (Figure 3(a)). She underwent TSS in 2004, where both adenomas were resected. The right PA was a sparsely granulated GH adenoma while the left PA corresponded to a prolactinoma, as confirmed by GH and prolactin staining on histological analysis (Figure 3(b)). Following surgery, she had two spontaneous pregnancies. She was discharged from the endocrine clinic, but when the potential genetic background of her disease was identified, she was recalled for genetic testing and the follow-up restarted after the *AIP* mutation detection. This patient had no contact with the proband's side of the family since the death of the proband's mother and family links were discovered via the genetic clinic (Figure 4). Fourteen years after her diagnosis, she has normal pituitary function and normal IGF-1 and prolactin, and her MRI is also normal. Her children are both asymptomatic carriers under paediatric endocrine follow-up.

## 6. Case 5

**Figure 5:**
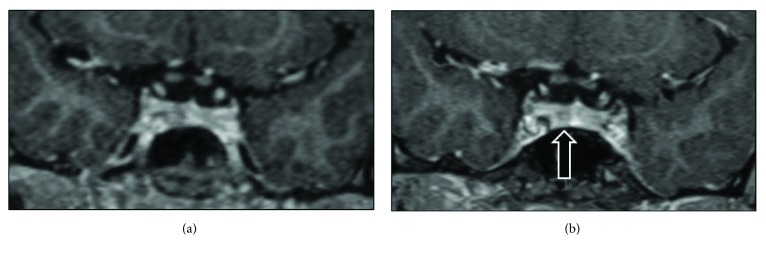
Pituitary MRI scans of the proband's first cousin (case 5), showing a normal pituitary at the age of 9 (a), and three years later, a 3 mm microadenoma became visible in her pituitary gland (b).

Following the genetic cascade screening, the proband's first cousin once removed was identified at the age of 7 years as an *AIP* mutation carrier. Follow-up was started at a paediatric endocrine clinic. She had no clinical symptoms and displayed a normal growth profile. At her first evaluation, her height was 124.8 cm corresponding to the 76th height centile (midparental height: 50th centile). Her baseline IGF-1 and prolactin were normal (Table 3). At the age of 9, she started complaining of headache partially refractory to analgesia and which affected her concentration and school work. A pituitary MRI excluded any pituitary lesion (Figure 5(a)). Her repeated hormonal profile was normal (Table 3). There is a family history of migraine.

Three years later (in 2017), at the age of 12 years, the headache worsened and an MRI scan showed a 3 mm area of reduced enhancement on the right side of her pituitary consistent with a small PA (Figure 5(b)). There were some inflammatory changes in the left sphenoid air sinus, which may be relevant given the history of headache. Concomitantly, her serum prolactin was raised at 784 mU/L (normal range < 496), and repeated prolactin measurements three months later, collected at 0 and 30 minutes after cannulation, showed again slightly raised prolactin levels (667 and 652 mU/L, resp.). Macroprolactinemia was excluded by polyethylene glycol precipitation, and there are no other apparent causes of hyperprolactinemia in this young patient. Of note, there is also a family history of constitutional delay of growth and puberty in her noncarrier 15-year-old brother who is under investigation by a paediatric endocrinologist. Her latest auxology and biochemical GH axis assessment at the age of 13.0 years is normal (Table 3). Her current height is 151.4 cm (SDS: −0.6), and her height velocity is normal (5.0 cm/year). Her thyroid function tests were normal. She is now in early puberty (Tanner stage A2 B2 P4). In view of her inherited predisposition for PA, this lesion likely represents a microprolactinoma. However, since she has now commenced puberty and her prolactin is only marginally raised and stable, dopamine agonist treatment has not been commenced. However, this will be reconsider if there is biochemical or radiological evidence of tumour progression or abnormal pubertal development.

## 7. Genetic Screening of This Kindred

At the time of the diagnosis of case 2 in 2002, MEN1 testing was performed, although the phenotype was not typical. Carney complex was ruled out based on clinical grounds. Following the identification of *AIP* mutations as predisposing for PAs in 2006 [7], conventional exon-exon sequencing of the *AIP* gene revealed no mutations. However, multiplex ligation-dependent probe amplification (MLPA) was performed later [15], and a heterozygous *AIP* exon 2 deletion (c.(99+1_100-1)_(279+1_280-1)del) was found in both brothers, as previously reported [8, 9, 15]. This exon deletion theoretically results in the in-frame ablation of 60 amino acids (A34_K93del) that correspond to three quarters of the FKBP12-like domain in the AIP protein and probably results in significant protein folding abnormality, if the shortened abnormal RNA is not degraded by nonsense-mediated decay [8, 15].

Cascade genetic screening identified 8 carriers (Figure 4). Carriers underwent endocrine evaluation, and 2 of them (case 3 and case 5) were diagnosed prospectively with PA. Overall, of 14 members carrying germline *AIP* mutations in this kindred (3/14 obligate carriers), 5 have PAs and 6 are asymptomatic carriers undergoing follow-up (Figure 4). The PA penetrance in this kindred is estimated at 36% (5/14). The 5 members with PAs, 2 males and 3 females, were all diagnosed at an age < 30 years; 3 presented clinically and 2 were diagnosed prospectively. There is variability in PA phenotypes in this kindred: 2 clinically NFPA, 1 somatolactotrophinoma, 1 microprolactinoma, and a concomitant prolactinoma and somatotrophinoma. At present, three patients are in remission (case 1, case 2, and case 4), and two females with pituitary microadenomas and mild prolactin elevation are under follow-up (case 3 and case 5).

## 8. *AIP* Mutation Carriers

Six alive asymptomatic *AIP* mutation carriers are under follow-up in the endocrine or paediatric endocrine clinics (Figure 4). Two carrier children in the family (aged 11 and 8 years) show normal growth, IGF-I, and prolactin levels, and 3 adult carriers have normal clinical, biochemical, and MRI assessments. One carrier, the proband's 45-year-old male cousin, has no clinical symptoms and normal MRI, but his IGF-1 was seen once slightly raised at 260 nmol/L [94–252] with an OGTT GH nadir of 0.28 *μ*g/L, with the rest of his pituitary function being normal. One year later, his IGF-1 was found within normal range (215 nmol/L [94–252]). The proband's mother, an obligate *AIP* mutation carrier, was diagnosed with a spinal ependymoma and died at the age of 45. The proband's at-risk second cousin, untested for *AIP* mutation, died of Hodgkin's lymphoma.

## 9. Patient Comments

In order to gain some insight into the experiences of patients and carriers, we asked three patients (case 1, case 2, and case 5) and one unaffected carrier (subject III.b) questions about the impact of having this mutation.

Case 1 and case 2 raised three main issues:

1. *The Impact of Headaches*. Both patients reported headaches prior to the treatment. “I suffered daily headaches which were something I was unaccustomed to.”
2. Even after treatment, case 2 reports that the headaches have returned but he has found ways to manage this. “Over the past 15 years…daily headaches have returned and become worse over time….Rubbing of the temples and cold hands help manage the pain. I know these episodes pass with time and this gives me reassurance while experiencing them.”
3. *Improvement after Treatment*. In both cases, the patients report significant improvement after treatment. “Treatment … was excellent and the results almost instant.”
4. Following treatment, case 1 felt he was soon at “full physical capacity.” Case 2 noticed that after surgery, “lethargy was no longer an issue” and although the headaches persisted, they were “no longer a daily recurrence” and would only become intense “under high-pressure conditions such as underwater or during a flight.”
5. *Future Choices and Vigilance*. Overall, both patients agreed that, although they remain vigilant for symptoms, their previous diagnoses and genetic status will not affect the choices they make in the future. The patients felt like they “could not be in safer hands” and felt confident that the condition was “entirely treatable and manageable.”
6. Therefore, being equipped with knowledge about symptoms and having the support from their medical team, they do not have any significant concerns about the future “…knowing symptoms and the capacity to diagnose much quicker means future issues with passing the mutated gene on through the bloodline is no longer a concern.”
7. “…having these issues has not affected the choices I have made for my future…I have a good understanding of my own capabilities and boundaries.”

From the interview of subject III.b (unaffected carrier) and his 12-year-old daughter (case 5), two main themes emerged:

1. *Emotional and Behavioural Impact of Having a Genetic Mutation*. In terms of emotional impact, subject III.b was initially disappointed to find out that he was a carrier of the *AIP* mutation, but not surprised. He also indicated that this knowledge was beneficial. “It was pretty disappointing to find out, but much better to know than to not know.”
2. Case 5 had a similar reaction. Initially, she was worried, but this gave way to positive feelings related to the benefit of having information regarding the genetic predisposition. What is particularly positive is that over time, case 5 has come to terms with her genetic status. “At first I was worried and upset and I didn't really understand what it was…Overall I think it is good, so I can get tested and if I have any issues they will be discovered early in the process and hopefully any treatment will be smaller”. “I have got more used to it.”
3. In terms of behavioural impact, subject III.b felt that finding out that he was a carrier had very little impact on behaviour. “In some ways it has made me more aware/conscious of my health, but I wouldn't say it has driven too much behavioural change.”
4. However, once it was established that one of his children was a carrier (case 5), “the impact was more significant”. “Since my daughter was confirmed as a carrier, we have been more aware and conscious of symptoms that may be connected to the condition. For example, she suffers from headaches and we watch them much more carefully then we would otherwise.”
5. *Routine Monitoring*. Subject III.b commented on the “inconvenience of annual blood tests and the occasional scan,” but overall he felt that it was “not too demanding” and that it was important to “continue with the testing regime and make sure that any future generations of the family are informed and tested.”
6. However, subject III.b did remark that the “testing routine is quite upsetting for a young child.” This was confirmed by her 12-year-old daughter (case 5) who commented on her dislike for the blood tests and scans. However, even though she dislikes the routine monitoring, she understands how important it is.
7. “I understand the importance of the tests even if I don't like them…I am also getting better at managing the blood test.”

## 10. Discussion

This large English kindred illustrates some key aspects and management challenges in *AIP* mutation-positive FIPA. The investigation, treatment, and follow-up of each case presented a number of unique challenging scenarios, providing learning opportunities and insights into clinical practice and genetic counselling, raising also some important questions that we aim to answer based on published data and our own experience with this condition [8, 16].

### 10.1. Diagnosis

The diagnosis of PAs can be delayed and is frequently made incidentally [2]. The diagnosis of the proband's case was delayed for months, initially interpreted in the context of grief. The fact is that he was remarkably hypothyroid and hypoadrenal at diagnosis and at risk of a life-threatening adrenal crisis. In contrast, his brother presented with headaches, but his diagnosis was faster due to the PA family history. This highlights the importance of patient and family member education for symptoms and the family history value in pituitary diseases. Age at diagnosis is lower in the second generation of FIPA families likely due to an increase in patient education and not in genetic anticipation [17].

Case 3 and case 5 illustrate the challenges associated with prospective diagnosis in *AIP* mutation carriers. Considering the high frequency of pituitary incidentalomas in the general population, up to 14–22% in radiological and autopsy studies [2], it is difficult to establish whether these lesions would behave differently from sporadic incidentalomas [18].

### 10.2. Headache

Headache is a major problem in patients with PAs, particularly in acromegaly [19, 20]. The presence of headache depends on PA subtype, size, tumour activity, cavernous sinus invasion, and predisposition for headache [20]. Both macroadenomas and microadenomas are linked with this problem [19, 21, 22]. In Cushing's disease series, nearly 30% of patients with microadenomas had headaches [21], whereas headaches are reported in 50% of acromegalic patients with microadenomas [23]. Headache features are variable, but most cases have chronic headache (46%) or episodic migraines (30%) [20]. Hypophysectomy may improve headaches in half of the cases, but can also paradoxically exacerbate them in 15% [20], or have no effect [24]. Somatostatin analogues may improve acromegaly-associated headaches in up to two-thirds of the patients; dopamine agonists may improve headaches in 25% but also exacerbate them in 21% of cases [20, 25–27].

Unfortunately, the fist surgery, short-acting octreotide, or cabergoline did not resolve case 2's headache, which was improved after the second TSS. Case 3 and case 5 also suffered from headache, and it is unclear if this is attributable to the small PAs or coincidental. Headache is common in the general population, with more than 50% of adults experiencing headache [28]; thus, patients with PAs may well have headache unrelated to their PA.

### 10.3. Double Adenoma

Case 4 had two different PAs concomitantly consisting of two different cell types. The possibility for multifocal PAs must be taken into account at diagnosis and during follow-up of any patient with germline *AIP* mutations, as all the anterior pituitary cells are haploinsufficient for AIP and therefore at risk. Multifocal PAs are rare in sporadic settings but have been described in patients with the Carney complex or MEN1 [29, 30]. In a surgical series of 117 patients with PAs, only 3 cases (2.6%) had double PAs and 1 had concomitantly a somatotrophinoma and a silent mammosomatotroph adenoma, whereas in the other 2 cases, coexistent lactotroph and null-cell adenomas were noted. These 3 cases were negative for *AIP* or *MEN1* mutations [31]. Another series including 600 surgical cases reported only 4 cases with double PAs (0.7%), confirmed histologically as GH-secreting PA plus gonadotroph PA in 2 cases and concomitant somatotrophinomas in other 2 patients [32]. A series of autopsies report double adenoma prevalences ranging between 0.9 and 2% in subjects with PAs [31, 33]. Double PAs can have a similar hormonal profile, more commonly ACTH [34–36] or GH [32], but different hormone-secreting double PAs seem to occur more frequently [31, 37]. In a large series, MEN1 patients had more frequently multiple PAs in comparison to non-MEN1 patients (4% versus 0.1%) [38]. Apart from our case [9] (one in 144 *AIP* mutation-positive PA patients [8], prevalence 0.7%), double PAs have been reported in an *AIP* mutation-positive patient where no surgery was performed so histology is not available [39]. GH- and prolactin-cosecreting PAs due to *AIP* mutations are frequent (23.9% in our cohort [8]), but GH and prolactin secretion from 2 distinct PAs is unique in our case 4. Thus, multifocal PAs should raise suspicion for familial disease.

### 10.4. Rathke's Cyst

Case 3 displayed a small Rathke's cyst which regressed within 5 years. Rathke's cysts arise from remnants of the embryologic Rathke's pouch and are found in 12–33% of normal pituitary glands in routine autopsies [40], 2-3 times more frequent in women [41]. In a cohort of 29 patients with symptomatic Rathke's cyst managed with surveillance, spontaneous involution was described in 31% and headaches were resolved spontaneously in 5 out of 7 cases [42]. Case 3's headaches continued despite the Rathke's cyst regression, so we cannot attribute this symptom to Rathke's cyst. Rathke's cysts have not been described in patients with *AIP* mutations apart from our case 3 [8].

### 10.5. Size of the Pituitary Gland in Teenager Females

Another challenge raised by case 3 is the pituitary volume in teenagers, especially females. Pituitary volume is normally increased in adolescents and young women. Neuroradiological series reported that 25–50% of healthy young women have a convex superior pituitary contour, and pituitary height more than 7, 8, or 9 mm can be found in 6.2%, 1.1%, and 0.5%, respectively [43].

### 10.6. GH Excess Diagnosis in Adolescence

GH excess diagnosis is challenging in adolescence at the peak of pubertal growth and for girls also when hormonal contraceptives are used [44, 45]. GH suppression after OGTT is gender and pubertal stage specific, with higher GH nadir in puberty and in girls: a study reported that the GH nadir is highest in Tanner stage 2-3 girls (1.57 ng/mL) and lower in other pubertal stages (0.64 ng/mL) or in boys (0.50 ng/mL) [45]. These GH dynamic testing cut-offs are different from those in adults [46]. Lack of GH nadir cut-offs on OGTT according to sex and pubertal stage makes the practical use of this test limited in children and adolescents. Moreover, delayed or accelerated puberty can result in a mismatch between the measurable IGF-1 and the chronological IGF-1 reference range [47]. The recognition of oral contraceptive use in females carrying *AIP* mutations is important for the interpretation of IGF-1 results, as they interfere with IGF-1 levels. Oestrogens lower IGF-1 levels, which explains why females have lower IGF-1 than men [48]. In women with mild acromegaly, oral contraceptives may normalise IGF-1 and reduce metabolic and body compositional effects of GH excess [48, 49]. In case 3, we found normal IGF-1. The effects on IGF-1, prominent with oral oestrogens, are minimal with transdermal formulations [50]; therefore, we exclude the influence of contraceptive patches (containing a combination of ethinyl oestradiol and norelgestromin) in her IGF-1 measurements.

### 10.7. Mild Abnormalities in Asymptomatic Carriers

One *AIP* mutation carrier in our kindred, the proband's male cousin (III.b), who has no symptoms of acromegaly and has normal MRI, was found with slightly increased IGF-1 (1.03 × ULN), but his GH nadir on OGTT was 0.28 *μ*g/L, and a repeated IGF-1 later was normal. Intermittent IGF-1 elevations, often with normal GH dynamic testing, with normal MRI have been seen in asymptomatic *AIP* mutation carriers in our cohort [8] and also reported by others [51]. Subtle abnormalities in the somatotroph axis in the absence of symptoms are typical in the Carney complex and related to somatotroph hyperplasia [52, 53].

In *AIP* mutation carriers, these mild biochemical abnormalities may be due to very small PAs not visible on MRI or due to somatotroph hyperplasia, described in a few *AIP* mutation-positive cases [54, 55]. Some of these cases do not fully fulfil the established diagnostic criteria for acromegaly and may represent a unique entity in the natural history of this condition.

### 10.8. Hyperprolactinaemia

The above considerations are valid for asymptomatic individuals carrying *AIP* mutations with mild hyperprolactinaemia, in whom its aetiology might be challenging to determine. Hyperprolactinaemia is common, due to physiological changes (stress, food ingestion, exercise, coitus, chest stimulation, sleep, lactation, and pregnancy), diseases (epilepsy, renal failure, cirrhosis, chest trauma, hypothyroidism, and polycystic ovary syndrome), or medications [56]. The relevance of mild hyperprolactinaemia, when macroprolactinaemia is ruled out, can be challenging when found concomitantly with a 3 mm PA, as in case 5. As hyperprolactinaemia may have significant inhibitory effects on puberty [57], careful follow-up is needed.

### 10.9. MRI Scanning during Carrier Follow-Up

Another point to consider in case 5 is the question of MRI scanning in paediatric settings. We usually suggest to perform the first MRI in *AIP* mutation carriers, if clinical and biochemical assessments are previously normal, at the age of 10 when children are reasonably expected to undergo an MRI without sedation [16]. The youngest known case with an *AIP* mutation and PA showed signs of tall stature at the age of 3 and was diagnosed with a macroadenoma with extensive extrasellar extension [58]. Our patient had the MRI at the age of 9, because of headaches, and showed no abnormality. MRI was repeated 3 years later due to worsening headache, and a pituitary microadenoma was seen. To our knowledge, this case is the first reported *AIP* mutation-positive patient presenting with a PA not seen on the first screening and emerging during imaging surveillance.

### 10.10. Extrapituitary Diseases

The proband's mother, an obligate *AIP* mutation carrier, died from a spinal ependymoma. There are reports in the literature of 11q13 abnormalities in ependymomas [59], and there is also a report of a MEN1 patient (*MEN1* is located at 11q13, like *AIP*) with an ependymoma [60]. Ependymoma has not been described in other *AIP* mutation-positive FIPA families [8]. We do not have access to the spinal ependymoma tissue; thus, we could not study the expression or loss heterozygosity to affirm an association with the *AIP* mutation. In our AIP mutation-positive patient cohort, we have eleven cases with extrapituitary neoplasms: two females with breast cancer, three cases with gastrointestinal stromal tumour (two in the same family), two related cases with meningioma [61], one with non-Hodgkin's lymphoma, one male with osteosarcoma and a colon neuroendocrine tumour [8], one with glioma (loss of heterozygosity of the AIP mutant locus was not present [62]), and a 21-year-old female carrier with a lipoma (unpublished). LOH at 11q13 with loss of *AIP* expression has also been reported in an adrenocortical carcinoma in a patient affected by a germline *AIP* mutation [63], but Chr11q loss of heterozygosity is common in adrenocortical cancers [8]. Other anomalies have also been detected in *AIP* mutation carriers, such as hydrocephalus and aneurysms [64].

### 10.11. PA Features in *AIP* Mutation-Positive Patients

In *AIP* mutation-positive kindreds, GH-secreting (45.7%) and GH- and prolactin-cosecreting (23.9%) PAs are the most common types [8]. Gigantism is particularly frequent, representing over a third of *AIP* mutation-positive patients [8, 10]. Variability in PA subtypes is seen in our kindred. *AIP* mutation-positive patients typically have PAs in the second decade (mean age at diagnosis between 18 and 24 years), and almost all are diagnosed before the age of 40 [8, 65, 66]. In our kindred, four cases were diagnosed before the age of 18, and case 4 was diagnosed at the age of 27 when stopping an oral contraceptive, so it might have been causing symptoms earlier. Patients with PAs associated with *AIP* mutations present earlier in life in comparison with sporadic or *AIP*-negative FIPA cases and tend to have more aggressive disease. However, not all *AIP* mutation-positive FIPAs have a rapidly growing and aggressive phenotype. In this kindred, the PAs were not particularly aggressive.

#### 10.11.1. What Is the Penetrance of *AIP* Mutation-Positive FIPA Kindreds?

Analysis of large kindreds suggested a heterozygous autosomal dominant inheritance pattern with incomplete penetrance in FIPA due to *AIP* mutations [7]. The penetrance of PAs in the kindred here reported is 36%, with 5 PA cases out of 14 members harbouring *AIP* mutations. Penetrance of PAs has been estimated to range between 15 and 30% [10]. Based on 3 extensively screened large families in our cohort, we found a penetrance of 22.7%, but when cases diagnosed prospectively were excluded, the penetrance was only 12.5%, highlighting the relevance of screening for apparently unaffected *AIP* mutation carriers [8]. The published data suggest that the mutation type (truncating versus missense) does not influence the overall penetrance, although it might influence the age of penetrance [8]. A germline *FGFR4* variant was excluded as a possible disease-modifying-disease gene, and *GNAS* mutations are not present in *AIP* mutation-positive PAs [8]. Variable penetrance could be explained by other, currently unknown, genes influencing disease manifestation, as well as by other factors, such environmental influences [51, 67, 68].

#### 10.11.2. Which Patients with PAs Should Be Tested for *AIP* Mutations?

No consensus guidelines in terms of genetic screening for *AIP* mutations and surveillance and management of *AIP* mutation asymptomatic carriers are available yet in this setting. Rather, expert recommendations are available on the basis of the literature. Patients with PAs that should be tested for *AIP* mutations include as follows:

1. Patients with a diagnosis of FIPA, that is, patients with a PA who have a family history of PA and do not have clinical or genetic features of any syndromic disease. Approximately 20% of all FIPA families have *AIP* mutations, and this rises to 40% if members have only somatotrophinomas and to almost 100% if two or more members have childhood-onset somatotrophinomas [10].
2. Patients with macroadenomas diagnosed before the age of 30, particularly those secreting GH and/or prolactin, who do not have an apparent family history of PAs. A significant proportion of these, approximately 12%, has *AIP* mutations [13, 69]. The lack of family history can be due to limited family history information, relatively low penetrance of PAs in *AIP* mutation carriers, or de novo *AIP* mutations [16], although only two cases of de novo mutations have been reported [70, 71].
3. Patients with a PA diagnosed before the age of 18 years. Up to 20% of these, usually somatotrophinomas or prolactinomas, have an *AIP* mutation. In paediatric Cushing's disease or NFPA, the chances of finding *AIP* mutations are lower, but still genetic testing is advocated [11, 13, 16, 71].
4. Patients with multifocal PAs, particularly in the case of GH and/or prolactin hypersecretion. Double PAs have been only reported twice in the *AIP* mutation-positive setting. Until more data is available, it is reasonable to test patients with double or multiple PAs for *AIP* mutations, particularly in the absence of syndromic features.

#### 10.11.3. How to Manage AIP Mutation-Positive Patients and Carriers?

1. Patients with FIPA due to *AIP* mutations who present clinically should be treated as any sporadic PA depending on their PA subtype and disease extension in accordance to current guidelines [18, 46, 72–74]. However, these tumours can be larger and more invasive and often respond poorly to therapy, requiring multimodal approach [10].
2. First-degree family members (parents, children, and siblings) of FIPA probands should be offered genetic testing for their familial *AIP* mutation following genetic counselling. In individuals with germline *AIP* mutations but no family history, referral of first-degree relatives is appropriate for cascade screening.
3. *AIP* mutation-positive individuals require genetic counselling. Autosomal dominant inheritance, available data on PA penetrance, pituitary disease manifestations, the need for clinical screening and follow-up, and the carrier AIP status of their children should be explained to patients and carriers.
4. *AIP* mutation carriers should have baseline testing including clinical examination, biochemical testing, and pituitary MRI. Prolactin and IGF-1 should be measured routinely, and in selected cases, GH on OGTT is required. Although the disease usually starts towards the second part of the first decade, there are cases reported earlier [58]. We recommend genetic screening by the age of 2 years, as other groups [10]. *AIP* mutation carriers should be assessed in terms of height and weight, height velocity, pubertal development, and baseline pituitary tests. Clinical assessment might start at the age of 3 years. Prolactin measurement should start early, since growth retardation is present only in a minority of children with prolactinomas [75, 76], thereby limiting the sensitivity of auxological evaluation, which is of utmost importance for early detection of GH excess. Thus, we recommend that prolactin and IGF-1 measurements should start by the age of 3 years and be repeated routinely on a yearly basis. In young children, unless biochemical or clinical evidence of disease, baseline MRI can be delayed until old enough to be performed without anaesthesia, usually 10 years, also because most childhood-onset cases start above this age [64].
5. *AIP* mutation carriers who are found to have clinical, hormonal, and radiological signs of PAs at baseline assessment should be treated according to the current guidelines [18, 46, 72–74].
6. Family members who are found to be *AIP* mutation carriers but do not have clinical, hormonal, or radiological signs of PAs at baseline assessment should be followed with clinical and biochemical assessment. Children and young adults can be evaluated clinically and biochemically on a yearly basis and instructed to seek assistance before any signs of pituitary disease. Follow-up pituitary MRI can be repeated every 5 years. Duration of regular screening is currently an open question. The majority of PAs are diagnosed before the age of 30–40 years [11]; so, it is conceivable to decrease and even stop screening frequency beyond this age if routine evaluations are consistently normal. Ending the follow-up can be considered in older patients, given the low probability of detecting PAs after the fifth decade of life [8], although this is not our current practice. Our oldest *AIP* mutation-positive patient presented with apoplexy due to large macroadenoma at the age of 66 years, while the oldest *AIP* mutation-positive patient was identified prospectively with a PA at the age of 69 years.

#### 10.11.4. How to Test for *AIP* Mutations?

More than one hundred *AIP* gene variants have now been described, including insertions/deletions, single-nucleotide polymorphisms, nonsense and missense mutations, duplications, promoter and splice site mutations, and large genomic deletions. Most disease-causing mutations lead to truncated protein (around 70%), and a few hotspots are identified to date (R304^∗^, R271W, and R81^∗^) [8]. Sequence analysis of *AIP* exons, exon-intron junctions, and promoter region will detect approximately 90% of *AIP* mutations. However, 10% of abnormalities in the *AIP* gene in FIPA families are due to large deletions, like in our kindred, which can be detected by MLPA [15, 16]. Therefore, *AIP* gene sequence analysis followed by MLPA is the recommended testing method.

When a rare and/or a new *AIP* variant is detected, careful assessment should be taken to understand if that variant is likely to cause disease or it might be a nonpathogenic variant, as this is of major importance for genetic counselling and for deciding whether family members require genetic screening. There are diverse forms to predict the pathogenicity of an *AIP* variant, such as segregation of that variant with the disease, its frequency in the general population, in silico predictions, *in vitro* functional studies, loss of heterozygosity demonstration, and conservation across species [64]. Both the International Agency for Research on Cancer (IARC) and the American College of Medical Genetics and Genomics (ACMG) suggest a 5-tier system to characterise variants: pathogenic, likely pathogenic, uncertain significance, likely benign, and benign [77, 78].

#### 10.11.5. Benefits of *AIP* Genetic Testing

Genetic and clinical screening has been shown to identify an unexpectedly high proportion, 20–25% of apparently unaffected carriers with PAs [8]. Genetic test for *AIP* mutations in individuals at risk and subsequent clinical screening in apparently unaffected *AIP* mutation carriers can result in early diagnosis and treatment of possibly aggressive PAs. Family members who do not carry *AIP* mutations will be relieved from follow-up. Psychological impact of long-term surveillance needs to be considered, and *AIP* mutation carriers should be reassured of the benignity and low penetrance of PAs but understand the benefits of early detection in case of disease. The lack of compliance with the long-term follow-up is often an issue, partially due to the low penetrance and benign nature of the disease.

## 11. Conclusions

The clinical and genetic characteristics of FIPA patients have been studied extensively over the last years. Current data suggest that genetic screening for *AIP* mutations will reveal on average 20% of FIPA families harbouring an *AIP* mutation, but the detection range is wide depending on the specific studied population, varying from 3% in unselected populations to almost 100% if two or more childhood-onset somatotrophinomas are identified in the same family. Prospective screening of family members can identify presymptomatic disease. Further research is needed to identify novel genes causing FIPA. The genetic counselling and clinical management of FIPA cases require a multidisciplinary approach which hopefully will improve prognosis and outcomes in the future. The kindred described here aid us to understand some of the clinical, diagnostic, and management challenges associated with *AIP* mutation-positive PAs, and it allows us to review essential aspects associated with the genetic diagnosis and screening of this setting.

## Figures and Tables

**Table 1:** Biochemical evaluation of the somatotrope and prolactin axes in the proband's brother (case 2), at diagnosis, postoperatively, and during follow-up. TSS: transsphenoidal surgery; ×ULN: times above the upper limit of normal.

Date of biochemical evaluation	IGF-1	Normal range	Prolactin	Normal range	GH on OGTT 0′–30′–60′–90′–120′–150′
February 2002 *Preoperatively*	**75.3** *(1.2 × ULN)*	29–64 nmol/L	**927**	45–375 mU/L	2.0–0.4–0.8–0.5–0.5 *μ*g/L
August 2002 *Six months postoperatively*	34.7 *(0.5 × ULN)*	29–64 nmol/L	239		
2002–2009	Results not available				
May 2009	317 *(0.8 × ULN)*	117–358 ng/mL	193	<496 mU/L	1.4–1.0–0.82–0.74–1.4–2.4 *μ*g/L
October 2009 *On short-acting octreotide (100 μg three times a day)*	266 *(0.7 × ULN)*	117–358 ng/mL	261	<496 mU/L	0.96–1.1–1.2–1.2–1.3 *μ*g/L
November 2010	**389** *(1.1 × ULN)*	117–358 ng/mL	298	<496 mU/L	
December 2010	168 *(0.5 × ULN)*	117–358 ng/mL	260	<496 mU/L	
July 2011	348 *(1.0 × ULN)*	117–358 ng/mL	256	<496 mU/L	
December 2012	326 *(0.9 × ULN)*	117–358 ng/mL		<496 mU/L	1.9–1.36–1.12–1.24–1.45–1.59 *μ*g/L
May 2014	**398** *(1.3 × ULN)*	125–302 *μ*g/L	297	<496 mU/L	
October 2015	**303** *(1.0 × ULN)*	125–302 *μ*g/L	313	<324 mU/L	
October 2016	**326** *(1.4 × ULN)*	82.5–240.4 *μ*g/L	**353**	<324 mU/L	3.41–3.17–2.53–2.59–2.91–2.87 *μ*g/L GH day curve over 10 hours 3.12 *μ*g/L, 1.93 *μ*g/L, 2.05 *μ*g/L, 3.23 *μ*g/L, 2.61 *μ*g/L. Mean GH = 2.59 *μ*g/L
January 2017 *On lanreotide*	**320** *(1.3 × ULN)*	82.5–240.4 *μ*g/L			
February 2017 *On lanreotide*	**280** *(1.2 × ULN)*	82.5–240.4 *μ*g/L			
August 2017 *Two months after the second TSS*	112 *(0.5 × ULN)*	82.5–240.4 *μ*g/L			
September 2017	93 *(0.4 × ULN)*	82.5–240.4 *μ*g/L	161	<324 mU/L	0.41–0.34–0.32–0.30–0.25–0.41 *μ*g/L

**Table 2:** Biochemical evaluation of the somatotrope and prolactin axes in the proband's sister (case 3).

Date of biochemical evaluation	IGF-1	Normal range	Prolactin	Normal range	GH on OGTT 0′–30′–60′–90′–120′–150′
November 2008	433	94–506 ng/mL	405	<496 mU/L	
August 2009	261	94–506 ng/mL	444	<496 mU/L	
February 2010	283	94–506 ng/mL	456	<496 mU/L	1.1–0.35–0.21–0.16–0.83–5.81 *μ*g/L
September 2010	164	94–506 ng/mL	329	<496 mU/L	
June 2011	333	94–506 ng/mL	349	<496 mU/L	
September 2012	172	94–506 ng/mL	312	<496 mU/L	1.07–0.35–0.22–0.19–0.28–1.05 *μ*g/L
October 2013	253	149–332 *μ*g/L	408	<496 mU/L	7.28–1.65–0.69–0.32–0.27–0.25 *μ*g/L
July 2015	224	149–332 *μ*g/L	365	<496 mU/L	10.56–1.65–0.73–0.38–0.39–0.62 *μ*g/L
December 2016	166	103.3–328.4 *μ*g/L	**601**	<496 mU/L	4.62–2.10–0.76–0.46–0.22–0.14 *μ*g/L

**Table 3:** Biochemical evaluation of the somatotrope and prolactin axes in the proband's second cousin (case 5).

Date of biochemical evaluation	IGF-1	Normal range	Prolactin	Normal range	GH on OGTT 0′–30′–60′–90′–120′–150′
March 2012	193	53–300 ng/mL	424	<496 mU/L	
March 2013	169	80–244 ng/mL	409	<496 mU/L	
March 2014	193	87–399 ng/mL	421	<496 mU/L	
June 2014	254	87–399 ng/mL	**509**	<496 mU/L	
January 2015	234	87–399 ng/mL	406	<496 mU/L	
January 2016	36	94–506 ng/mL	391	<496 mU/L	
November 2016			**784**	<496 mU/L	
February 2017	232	101–576 *μ*g/L	**667** (0′) **652** (30′)	<496 mU/L	1.80–0.79–0.28 (60′)–0.19 (120′)–2.03 *μ*g/L
November 2017	346	101–576 *μ*g/L	**675**	<496 mU/L	

## Additional Points

*Patient Comments*. Minor amendments were made to patient comments for clarity.
